# Database of space life investigations and bioinformatics of microbiology in extreme environments

**DOI:** 10.3389/fmicb.2022.1017773

**Published:** 2022-11-03

**Authors:** Junyong Wang, Tao Wang, Xian Zeng, Shanshan Wang, Zijie Yu, Yiqi Wei, Mengna Cai, Xin-Yi Chu, Yu Zong Chen, Yufen Zhao

**Affiliations:** ^1^Qian Xuesen Collaborative Research Center of Astrochemistry and Space Life Sciences, Institute of Drug Discovery Technology, Ningbo University, Ningbo, China; ^2^Department of Biological Medicines & Shanghai Engineering Research Center of Immunotherapeutics, Fudan University School of Pharmacy, Shanghai, China; ^3^Institute of Civil Design, Tsinghua University, Beijing, China; ^4^The Key Laboratory for Chemical Biology of Fujian Province, Department of Chemical Biology, College of Chemistry and Chemical Engineering, Xiamen University, Xiamen, China; ^5^Key Laboratory of Bioorganic Phosphorus Chemistry and Chemical Biology (Ministry of Education), Department of Chemistry, Tsinghua University, Beijing, China

**Keywords:** spaceflight, space life investigation, microbiology, database, bioinformatics

## Abstract

Biological experiments performed in space crafts like space stations, space shuttles, and recoverable satellites has enabled extensive spaceflight life investigations (SLIs). In particular, SLIs have revealed distinguished space effects on microbial growth, survival, metabolite production, biofilm formation, virulence development and drug resistant mutations. These provide unique perspectives to ground-based microbiology and new opportunities for industrial pharmaceutical and metabolite productions. SLIs are with specialized experimental setups, analysis methods and research outcomes, which can be accessed by established databases National Aeronautics and Space Administration (NASA) Life Science Data Archive, Erasmus Experiment Archive, and NASA GeneLab. The increasing research across diverse fields may be better facilitated by databases of convenient search facilities and categorized presentation of comprehensive contents. We therefore developed the Space Life Investigation Database (SpaceLID) http://bidd.group/spacelid/, which collected SLIs from published academic papers. Currently, this database provides detailed menu search facilities and categorized contents about the studied phenomena, materials, experimental procedures, analysis methods, and research outcomes of 448 SLIs of 90 species (microbial, plant, animal, human), 81 foods and 106 pharmaceuticals, including 232 SLIs not covered by the established databases. The potential applications of SpaceLID are illustrated by the examples of published experimental design and bioinformatic analysis of spaceflight microbial phenomena.

## Introduction

The development of space-based technologies has enabled extensive life science investigations (Afshinnekoo et al., 2020) on space platforms such as International Space Station (ISS, all the abbreviations can be found in Supplementary Table S1; Witze, 2020), space shuttles, and satellites (Afshinnekoo et al., 2020). In particular, various experiments have revealed distinguished effects of the space environment on microbial growth (Kim et al., 2013), colonization (Checinska Sielaff et al., 2019), survival (Yamagishi et al., 2018; Kawaguchi et al., 2020), metabolite production (Benoit et al., 2006; Huang et al., 2015), biofilm formation (Zhao et al., 2019), virulence development (Hammond et al., 2013; Gilbert et al., 2020), and drug resistant mutations (Fajardo-Cavazos and Nicholson, 2016). These effects offer useful perspectives to microbiology research. Some of these effects have been exploited for microbial production of pharmaceuticals, food components, and industrial metabolites in space (Cortesão et al., 2020). The impact of spaceflight on life occurs across multiple scales and systems. The relevant knowledge is both important for spaceflight investigations and provides useful perspectives to ground-based research such as microbiology. The investigations of the complex spaceflight effects require interdisciplinary efforts and integration of information resources across multiple fields (Goswami et al., 2013).

Space life science investigations (SLIs) and subsequent studies can be facilitated by the establishment of several specialized databases (Table 1). NASA Life Science Data Archive (LSDA)^1^ provides brief reports of 2,659 SLIs mainly founded by NASA. Erasmus Experiment Archive (EEA)^1^ contains brief reports of 4,187 European Space Agency (ESA) funded or co-funded spaceflight experiments (2,550 SLIs). NASA GeneLab database (Berrios et al., 2021) includes 359 SLI omics datasets. The entries in these databases can be searched and browsed by categories like species, research field, etc. These categories were summarized in Supplementary Table S2. Some databases focus on specific areas or missions. For example, Lifetime Surveillance of Astronaut Health database,^1^ which integrated in LSDA, provides 51 sets of clinical test data of astronaut occupational surveillance. The developing Integrated Biobank for Space Life Science^2^ currently contains 4 available sets of omics data from mouse habitat missions in ISS conducted by Japan Aerospace Exploration Agency (JAXA).

**Table 1:** Comparison of SpaceLID and established space life science databases.

Databases	LSDA	EEA	GeneLab	SpaceLID
Number of SLIs	2,659	2,550	359	448
Data sources	NASA funded studies Data submitted by users	1. ESA funded studies	Data uploaded by the creators Data submitted by users	1. Published academic journal papers
Data types	Experimental procedure and outcomes Experimental datasets Experimental related publications	Experimental procedure and outcomes Experimental datasets Experimental related publications	Raw and metadata of omics studies Environmental data Data related publications.	Experimental procedure and outcomes, shown in figures and tables Publications
Analysis tool	No	No	Datamining and visualization tools	No
Data categorization^*^	11 primary categories, 537 sub-categories.	5 primary categories, 504 sub-categories.	6 primary categories, 156 sub-categories.	6 primary categories, 15 sub-categories, 423sub-sub-categories.

^*^ Detailed introduction of data categorization can be found in Supplementary Table S2. The categorizes of each database can be achieved in the following links: LSDA: https://lsda.jsc.nasa.gov/Experiment; EEA: https://eea.spaceflight.esa.int/portal/?; GeneLab: https://genelab-data.ndc.nasa.gov/genelab/projects; SpaceLID: http://bidd.group/spacelid/browse.php.

There is an increasing interest of SLIs across diverse fields, both for spaceflight and ground-based life investigations. In particular, microbial investigations from different aspects have been performed in spaceflights such as: cell biology investigation of the effects of microgravity and nutrition on microbial growth (Kim et al., 2013), system biology study revealed microbial microgravity response pathways (Roy et al., 2016), and studies reported genomic and transcriptomic changes during spaceflight (Li et al., 2014; Morrison et al., 2019). Several studies also showed industrial and medical implications of space environment, such as the production of useful secondary metabolism (Benoit et al., 2006; Huang et al., 2015), the virulence change of pathology (Hammond et al., 2013), the enhancement of antibiotic resistance (Fajardo-Cavazos and Nicholson, 2016), and hardware design studies (Rabbow et al., 2017). To better facilitate broad research interests across diverse fields, there is a need for databases equipped with search facilities convenient to non-experts and information contents that are both comprehensive and categorized for efficient evaluation.

We therefore developed a new database, Space Life Investigation Database SpaceLID,^3^ which records investigations published in peer reviewed academic journals. In SpaceLID, each publication constitutes an entry, which commonly includes the procedure and outcomes presented in figures and tables of multiple experiments. In comparison, LSDA and EEA entries mainly constructed based on reports of single experiments, including descriptions of the procedure and outcomes mainly in text^1^; while GeneLab focus on sharing omics data (Table 1). To a certain extent, the entries of SpaceLID present the protocols of SLIs more comprehensively and display their outcomes more intuitively. For providing detailed menu search facilities and categorized information, SpaceLID attached tags to individual SLIs, which summaries the multiple aspects of the investigations (Supplementary Table S2). Compared to the established databases, the tags in SpaceLID are more detailed and hierarchical in information presentation. These tags are also inclined to show the biological significance, which facilitate the identification of SLIs in specific fields. The potential applications of the SpaceLID information contents are illustrated by the literature-reported bioinformatic analyses of spaceflight microbial data.

## Materials and methods

### Data collection and processing

The contents of SpaceLID were obtained by the following procedure. First, literatures of SLIs were searched from PubMed (Sayers et al., 2022) using keyword combinations between spaceflight-related terms and life science-related terms (Table 2). For example, search “International Space Station microbe” return 63 results. Secondly, these publications were manually checked for selecting experimental SLIs to be included in Space-LID. The following types of publications were excluded in SpaceLID: (1) Not describing original experimental investigations (e.g., reviews, commentaries, news). (2) Without sufficient descriptions about the experiments performed in spaceflight, or works focused on spaceflight simulations (e.g., simulated microgravity). The remaining ones were then assigned their respective unique identification IDs in the format SLID-XXX. Thirdly, the selected publications were manually evaluated for summarizing categorized information. The generated categories reflect four class of information: (1) The materials be investigated, including species, tissue, and organs. (2) The methodology be used, including experimental setups, analysis methods, etc. (3) The focus of the publication, such as the studied phenomena and properties. (4) In addition to the information provided in the publication, the relevance between the study and certain fields, i.e., medicine and biophysics was also manually annotated. The categorized information of publications was attached to their IDs. Finally, the contents of publications were organized into plain text tables in a fixed format. The tables recorded the publication information (title, journal, doi), the tags summarized in the previous step, the spaceflight program, the research protocols, and the research outcome. The tables and figures in the investigation outcomes were regenerated from the origin file.

**Table 2:** List of key words searched in PubMed for finding space life investigations.

Spaceflight-related terms	Life science related terms
International Space Station, ISS, satellite, space shuttle, spaceflight	amphibian, astronaut, bacteria, bacterial, biofilm, biology, bird, blood, bone, brain, cardiovascular, drug, fish, food, fruit, fungal, fungus, health, human, leaf, liver, lizard, medicine, mice, microbe, microbial, microbiota, monkey, mouse, murine, plant, rat, rodent, root, seed, seedling, shoot, skeletal, vegetable, worm

### Database construction

The SpaceLID data entries were stored in MySQL (version 5.7.34) database management system, which is an efficient and popular relational database management system. In order to be compatible with users and improve presentation, the front-end of web pages were constructed with HTML5, CSS3 and JavaScript. The interaction between the front-end and the database is achieved through PHP (version 7.2.24) which is an open source and efficient server-side programming language. SpaceLID was published using the Nginx (version 1.14.0) server with lightweight and free characteristics that could meets our needs.

The contents of SpaceLID can be searched by browse, keyword search, and menu search options. The browse page can be accessed by clicking the “Browse” button on the top panel of the SpaceLID main page. In the browse page, SLIs can be browsed by Kingdom of life (microbes, plants, human, and animals), space medicines, space foods, and space biophysics phenomena. Keyword search window is located in the center of the SpaceLID main page. Menu search panels are in the Advanced search section in the lower part of main page. The menu search panels include menus for organism, phenomenon (e.g., microbiome changes), project type (spaceflight, ground), research outcome type (outcome with categorizable data, outcome without categorizable data).

## Results

### SpaceLID database contents and access facilities

**Figure 1:**
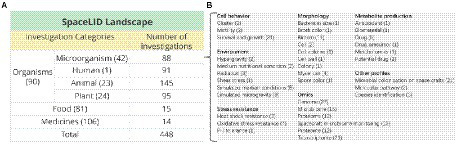
**(A)** Space Life Science Investigations (SLIs) in SpaceLID. The numbers in parentheses indicate the species, foods, and medicines recorded in the database. **(B)** The investigated phenomena or properties of the microbial SLIs. The number of SLIs in each filed is shown in the parentheses.

Our search procedure led to 448 literature-reported space life investigations (SLIs) with detailed information, in which 236 are not included in the established databases. Currently, SpaceLID cover 90 species. The landscape of SpaceLID is shown in Figure 1A. In particular, the 88 microbial SLIs involved in multiple fields of microbiology, such as biofilm, microbial colonization on space crafts, and microbial production of pharmaceuticals and metabolites (Figure 1B).

For the convenience of users of diversified backgrounds, the SpaceLID recorded SLIs are annotated with tags of different research fields (Supplementary Table S2). In the Browse page,^4^ the SLIs are sorted by organism category by default. The four primary categories are Microbe, Plant, Animal, and Human. According to the properties of the study in these categories, different tag classes were designed, as shown in Table 3. Click the tag classes can expand the corresponding selectable tag list. Click the “BROWSE” button, the studies with selected tags can be surmised into a table. For example, select “Metabolite production - pharmaceutical metabolite” and browse, seven SLIs can be found: such as antibiotics Actinomycin D produced by *Streptomyces plicatus* WC56452 (Benoit et al., 2006; Lam et al., 2006), Natamycin produced by *Streptomyces gilvosporeus* LK-22 (Liang et al., 2007), and Monorden produced by *Humicola fuscoatra* WC5157 (Lam et al., 1998; Supplementary Figure S1).

**Table 3:** Tag classes in SpaceLID.

Tag classes	Contents	Available primary categories
Project type	Types of the aircrafts (or ground study) used in the SLIs.	Microbe, Plant, Animal, Human
Investigated time	The time of the publication of the SLIs.	Microbe, Plant, Animal, Human
Profile	The investigated phenomenon, property, or other objectives of the SLIs.	Microbe, Plant, Animal, Human
Part	The biospecimen studied in the SLIs.	Plant, Animal, Human
Organism	The scientific and common name of the investigated species.	Microbe, Plant, Animal
Disease/Health condition	The associated diseases or conditions of the SLIs.	Microbe, Animal, Human
Microbe form	The form of microbes during spaceflight.	Microbe

The browse result is summarized in a table, in which each SLI contains six columns: the unique ID of the SLI in SpaceLID, the title of the SLI, the investigated species, the studied biological phenomenon, the associated disease condition, and the studied biophysical phenomenon (Supplementary Figure S1). The ID is a hyperlink to the corresponding page of each SLI. The study page provides the following information: (1) Study Description: all tags within the study and the abstract. (2) Spaceflight information: the flight program, duration, and the information link. (3) Protocols: The assay performed in the SLI and the brief introduction of the corresponding methods. (4) Study Outcomes: the main results of the SLI summarized in categorized boxes. The corresponding formatted figures, tables and assays are attached in the boxes. (5) Publications: the information about the paper. To facilitate the crosstalk between SLIs and other fields such as biophysics, pharmaceutical science, and food science, SpaceLID also support browse investigations by Space Biophysics Phenomenon, Space Medicines, and Space Foods.

### Application of SpaceLID for information of experimental design of antibiotics production by spaceflight microbes

The application of SpaceLID database can be illustrated by an example of the information for the experimental design of microbial production of antibiotics in space. By inputting “antibiotic production” in the SpaceLID keyword search window, one can find 4 studies of microbial production of antibiotics in space (Lam et al., 1998, 2006; Benoit et al., 2006; Liang et al., 2007). By clicking an entry SLID-305, one can access the information page of spaceflight production of the antibiotic drug Actinomycin D by *Streptomyces plicatus* WC56452 (Benoit et al., 2006). From the Experimental or treatment protocol section, one can find useful information for the design of a spaceflight experiment in microbial production of antibiotics. This experiment can be conducted with a hardware called Multiple Orbital Bioreactor with Instrumentation and Automated Sampling (MOBIAS), which has been designed for long-term cell culture growth on ISS through semi-continuous fed batch processing.^5^ The MOBIAS hardware can be inserted into the Commercial Generic Bioprocessing Apparatus (CGBA) for thermal and process control.^6^ The microbial cultures can be maintained in three phases throughout the spaceflight. The first phase involves the processes from sample loading to orbit detection with the CGBA temperature held at 10.2 ± 0.6°C to prevent significant culture growth prior to microgravity exposure. The second phase consists of batch mode culture growth, with the CGBA temperature increased over a period of ~45 min and maintained at 22.0 ± 0.2°C for the duration of spaceflight. The third phase transitions the culture to fed-batch conditions to provide fresh medium and allow for the removal of spent medium and byproducts to maintain viable cultures for the long duration (e.g., 72-days) spaceflight.

### Application of SpaceLID for information of bioinformatic analysis of antimicrobial resistance and virulence genes of spaceflight microbes

A second example of SpaceLID application is the information for bioinformatic analysis of the antimicrobial resistance genes (AMR-genes) of spaceflight microbes. The search of SpaceLID by keyword “antimicrobial resistance” led to 3 entries of spaceflight antimicrobial resistance investigations. In particular, entry SLID-344 is about the detection of AMR-genes associated with the ISS environmental surfaces, where bioinformatic analysis has been conducted on 63 detectable AMR-genes of 21 BSL-2 bacterial strains from 24 samples collected from the ISS (Urbaniak et al., 2018). These 24 samples have been collected during 3 sampling events (flight F1, F2, and F3) at 8 locations onboard ISS, and tested for resistance against 9 antibiotics (cefazolin, cefoxitin, ciprofloxacin, erythromycin, gentamycin, oxacillin, penicillin, rifampin, and tobramycin). Because elevated AMR-genes indicate the levels of antimicrobial resistance, the abundance profiling of the 63 AMR-genes with respect to the flights and ISS locations may provide useful clues about the environmental conditions for constraining the abundance of AMR-genes, which is important for developing mitigation strategies to maintain astronaut health during long duration spaceflights.

**Figure 2:**
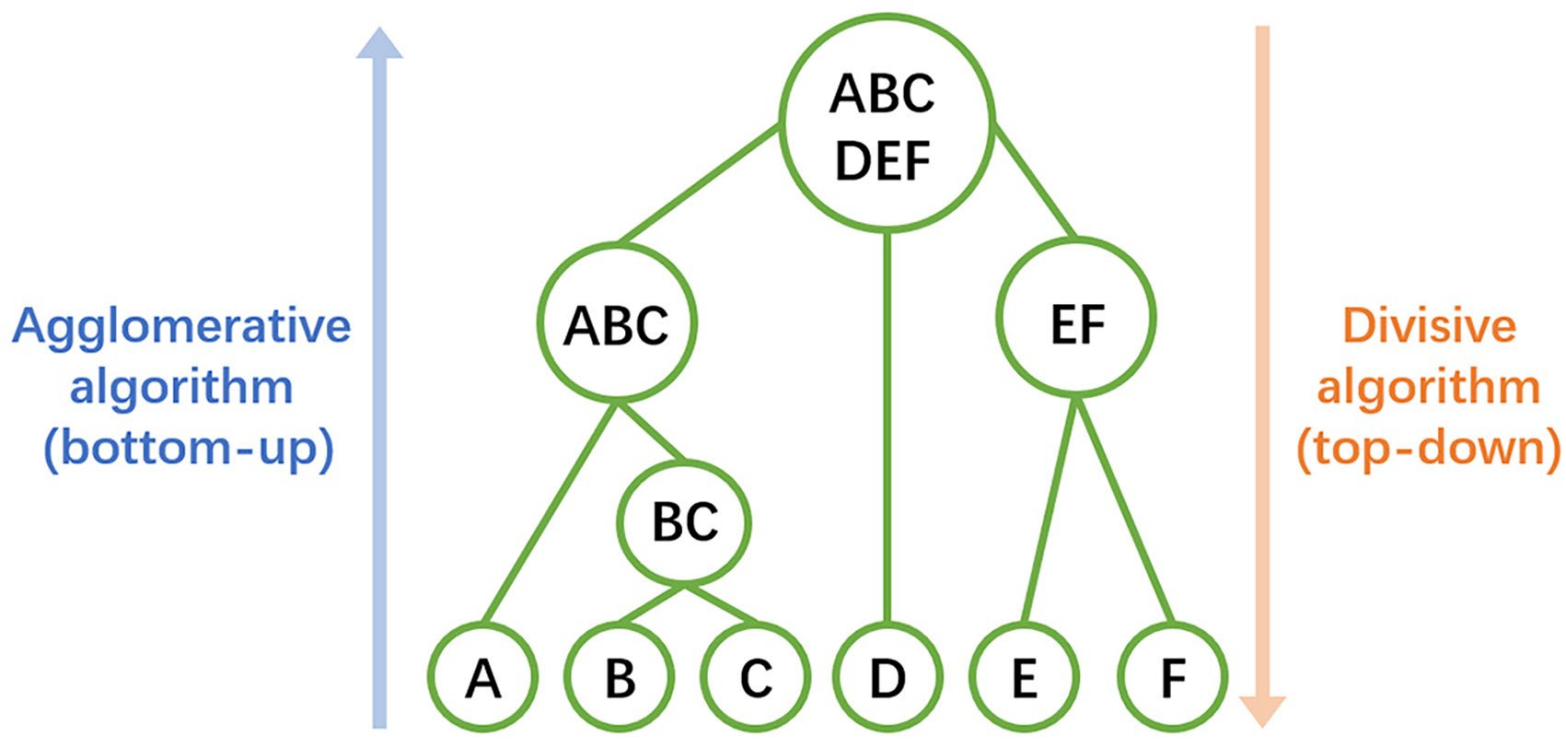
Illustration of hierarchical clustering method. Samples and classes are represented by the uppercase letters and circles. Agglomerative algorithm (bottom-up) firstly treats every individual sample as singleton classes, then merges pairs of classes successively, until all clusters merged into a single one. Divisive algorithm (top-down) firstly treats all samples as one class, then splitting the class recursively, until all individual samples split into singleton classes.

**Figure 3:**
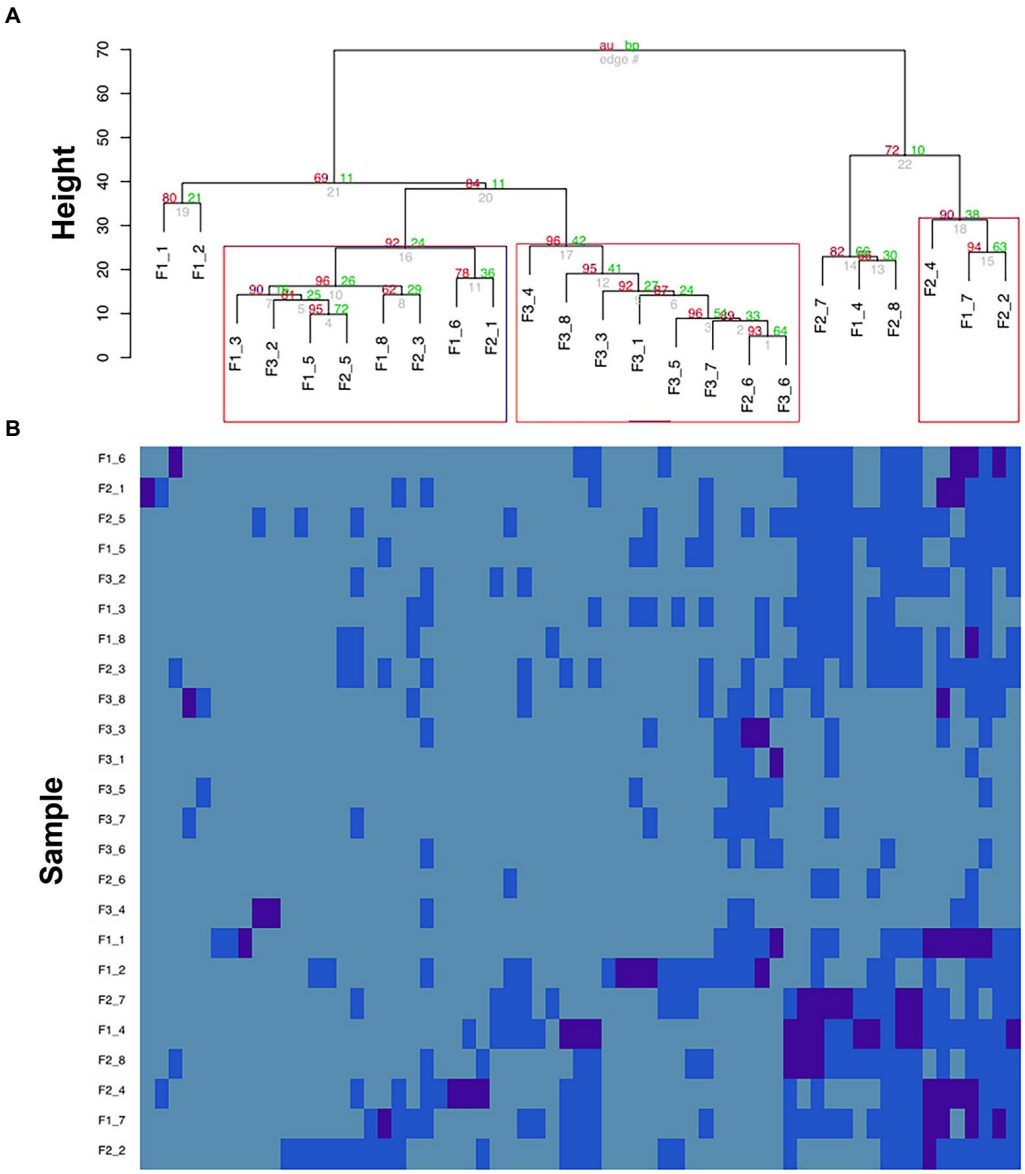
Temporal comparison of AMR-gene profiles (modified from a figure in Urbaniak et al., 2018). **(A)** Hierarchical clustering of the AMR-genes. Red values are AU (Approximately Unbiased) *p*-values, and green values are Bootstrap probability values. Clusters with AU p-values larger than 90% are highlighted by rectangles, meaning that there is a 90% certainty of these clusters being a distinct group. F“x” refers to the flight number, followed by the location. **(B)** Heatmap of centered log ratio transformed gene expression data. Deep blue boxes represent samples that have higher gene expression then the geometric mean expression (light blue), which has been calculated from all samples, which has been calculated from all samples.

The relative abundances of the AMR-genes can be displayed in a bar-plot by taking the read count of each AMR-gene in a sample and dividing by the total read count of that sample. The abidance profile of the 63 AMR-genes with respect to the flights and ISS locations can be analyzed by means of hierarchical clustering method (Thalamuthu et al., 2006), based on Euclidian distances of centered log ratio transformed gene abundance data. This analysis finds genes that share similar expression pattern in certain samples or treatments, which may have related functions or located within the same pathways. Hierarchical clustering can be performed by both agglomerative (bottom-up) or divisive algorithm (top-down), which are usually chosen according to the amount of data and the aimed number of classes (Figure 2). In the study as the example (Urbaniak et al., 2018), the clustering patterns of the 63 AMR-genes can be visualized by a dendrogram (Figure 3A) using the pvclust package^7^ and by a heatmap (Figure 3B) generated with the gplot package.^8^ These profiles reveal lower relative abundances of most of the 63 AMR-genes during the F3 flight with respect to the F1 and F2 flights. Further investigations of the environmental conditions, sanitary measures, and activities on F3 with respect to those on F1 and F2 may shed light on the factors that constrain the abundance of AMR-genes on spaceflights.

The usefulness of SpaceLID for the information of the spaceflight bioinformatics analysis can be illustrated by another example. By selecting “Virulence” tag under the “Disease/Health Condition” button of “Microbe” class in SpaceLID browse page, 10 entries were presented in the result page. Among these entries, SLID-155 is based on a study which applied shotgun metagenome sequencing to determine the virulence capabilities of the microbial communities separated from ISS samples (Singh et al., 2018). The sequencing data was analyzed by MEGAN6, a comprehensive toolbox for metagenome analysis (Huson et al., 2007). Filtered sequencing reads form each sample was clustered by the MEGAN6 equipped lowest common ancestor algorithm, a hierarchical clustering method assign reads to taxa. The organisms were then identified by searching the taxa reads in the NCBI taxonomy database using DIAMOND (Buchfink et al., 2015) and MEGAN6. The pathogenic organisms were annotated by the Bacterial and Fungal Risk Group Database.^9^ The sequencing reads were also translated to protein and then searched in references databases eggNOG (Powell et al., 2012), SEED (Overbeek et al., 2005), and KEGG (Kanehisa and Goto, 2000) to estimate their function. The virulence related reads were classified into categories based on the reference virulence factors supplied by the SEED database. Several virulence gene group such as multi-drug-resistant resistance efflux pump were found to be prevalent in samples. The SpaceLID entry SLID-155 recorded the detail methods and outcomes of this study.

### Application of SpaceLID for information of bioinformatic methods of molecular pathway analysis of spaceflight microbes

**Figure 4:**
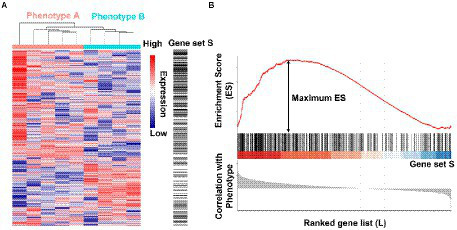
Illustration of the gene set enrichment analysis (GSEA) method. **(A)** A gene expression dataset sorted by correlation with phenotype, the corresponding heatmap, and the “gene tags,” i.e., location of genes from a set S within the sorted list. **(B)** Plot of the running sum for S in the data set, including the location of the maximum enrichment score (ES) and the leading-edge subset. The figures were drawn using data from GSE35958, only used to show the principle of GSEA method.

**Figure 5:**
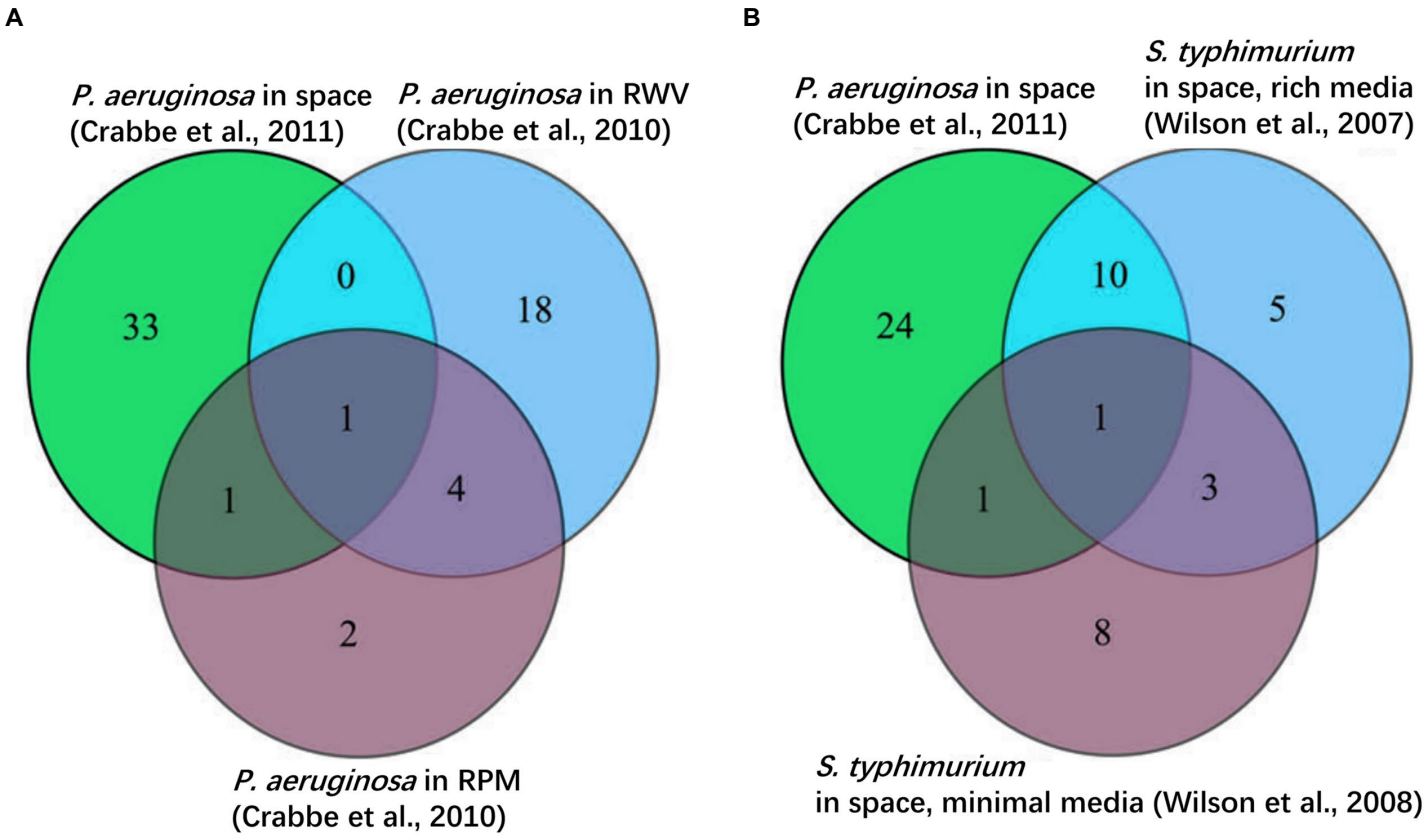
Venn diagrams among different experiments showing common altered pathways (modified from a figure of Roy et al., 2016). **(A)** *P. aeruginosa* in space, rotating wall vessel (RWV), and random positioning machine (RPM) experiments. Valine, leucine, and isoleucine degradation pathways are common among the three experiments. Flagellar assembly, protein export, ribosome, RNA degradation, TCA cycle, and oxidative phosphorylation are increased in RWV and RPM but decreased in space. **(B)** *P. aeruginosa* in space, *S. typhimurium* in space (rich media), and *S. typhimurium* in space (minimal media). Ribosome, RNA degradation, protein export, flagellar assembly, methane metabolism, toluene degradation, oxidative phosphorylation, TCA cycle, glycolysis, purine metabolism, and pyrimidine metabolism are the common altered pathways.

A third example of SpaceLID application is the information of bioinformatics methods for probing the molecular pathways of microbes altered in spaceflight. The search of SpaceLID by the keywords “molecular pathways” led to 4 SLI entries involving the analysis of molecular pathways of different species in space. Specifically, entry SLID-333 describes systems biology analysis for revealing the molecular pathways of two proteobacteria altered in spaceflight (Roy et al., 2016). The two proteobacteria are *Pseudomonas aeruginosa* PAO1 (*P. aeruginosa*) and *Salmonella enterica serovar Typhimurium* (*S. typhimurium*). The effects of spaceflight on these bacteria may be inspected by differential gene expression analysis. However, gene expression changes of bacteria are generally low under microgravity (Wilson et al., 2007). Hence, passive mapping of differentially expressed genes to pathways may not reveal statistically confident profiles of molecular pathways in spaceflight. To overcome this problem, the gene set enrichment analysis (GSEA) method (Subramanian et al., 2005) implemented in the GenePattern 2.0 package (Reich et al., 2006) may be employed for knowledge-based interpretation of the expression profiles of focused gene sets. Based on the prior annotation of the gene groups shared functions or other properties, through calculated the tendency of genes in certain set S close to an extreme (most up-or down-regulated) of all ranked genes L (represented by an Enrichment Score, ES), GSEA can determine whether these genes significantly correlated with the investigated phenotype (Figure 4). GSEA has revealed microbial cellular and metabolic pathways altered in spaceflight (Roy et al., 2016). As shown in the example study (Roy et al., 2016), the common pathways of the two proteobacteria altered by spaceflight may be displayed by Venn diagrams (Wilson et al., 2007; Wilson et al., 2008; Crabb et al., 2010; Crabbé et al., 2011), which reveals that the number of overlapping pathways between *P. aeruginosa* and *S. typhimurium* in space is significantly higher than the number between *P. aeruginosa* in space and simulated microgravity conditions (Figure 5). Examples of the commonly altered pathways between *P. aeruginosa* and *S. typhimurium* in space are ribosome, RNA degradation, protein export, flagellar assembly, methane metabolism, toluene degradation, oxidative phosphorylation, TCA cycle, glycolysis, purine metabolism, and pyrimidine metabolism. Further examinations of these pathways may shed lights on the common stress response of the microbes to spaceflight conditions.

## Discussion

The extreme environment of outer space has profound effects on life and human health in space (White and Averner, 2001; Checinska Sielaff et al., 2019). The knowledge of the response of life under space environment is not only important for space exploration (Afshinnekoo et al., 2020), but also benefit terrestrial applications (Ruyters and Stang, 2016; Maiwald et al., 2021). Investigations and knowledge of space microbiology is particularly important for space safety (Mermel, 2013; Bijlani et al., 2021), biotechnology development (Cockell, 2022), resource and waste recycling (Lindeboom et al., 2018). Technologies from diverse disciplines such as nanotechnologies, bioinformatics (Schmidt and Goodwin, 2013; Castro-Wallace et al., 2017) and artificial intelligence (AI; Scott et al., 2021; Madrigal et al., 2022) have been and are being applied for space life investigations. Established (LSDA, EEA, GeneLab, etc.) and newly emerged SLI databases can be enriched with expanding information and data of space investigations, and more convenient access facilities can be introduced by these databases. These databases can also provide useful information for facilitating the broad research interests across diverse fields and for the development of new enabling technologies in support of future space life investigations.

## Data availability statement

The original contributions presented in the study are included in the article/Supplementary material, further inquiries can be directed to the corresponding authors.

## Author contributions

JW, TW, and X-YC: investigation, methodology, and editing. JW and XZ: investigation, methodology, and validation. JW, SW, ZY, and YW: investigation and visualization. MC: supervision and validation. YZ: supervision and funding acquisition. YC: conceptualization, supervision, validation, writing—review, and funding acquisition. All authors contributed to the article and approved the submitted version.

## Funding

This study was supported by the Space Exploration Breeding Grant of Qian Xuesen Lab (TKTSPY-2020-04-03), Scientific Research Grant of Ningbo University (215-432000282), and Ningbo Top Talent Project (215-432094250).

## Conflict of interest

The authors declare that the research was conducted in the absence of any commercial or financial relationships that could be construed as a potential conflict of interest.

## Publisher’s note

All claims expressed in this article are solely those of the authors and do not necessarily represent those of their affiliated organizations, or those of the publisher, the editors and the reviewers. Any product that may be evaluated in this article, or claim that may be made by its manufacturer, is not guaranteed or endorsed by the publisher.

## Supplementary material

The Supplementary material for this article can be found online at: https://www.frontiersin.org/articles/10.3389/fmicb.2022.1017773/full#supplementary-material

## Footnotes

^1^ https://lsda.jsc.nasa.gov/

^2^ https://eea.spaceflight.esa.int/portal/

^3^ https://lsda.jsc.nasa.gov/LSAH/LSAH_Home

^4^ https://ibsls.megabank.tohoku.ac.jp/

^5^ http://bidd.group/spacelid/

^6^ https://lsda.jsc.nasa.gov/Experiment/exper/1796

^7^ http://bidd.group/spacelid/browse.php

^8^ https://lsda.jsc.nasa.gov/Hardware/hardw/1403

^9^ https://lsda.jsc.nasa.gov/Hardware/hardw/908

^10^ https://github.com/shimo-lab/pvclust

^11^ https://gplot.readthedocs.io/en/latest/basic.html

^12^ https://my.absa.org/Riskgroups
