# Supplementary material for: Database of space life investigations and bioinformatics of microbiology in extreme environments
Source: Front Microbiol. 2022 Nov 3;13:1017773. doi: 10.3389/fmicb.2022.1017773 (PMC9668873; doi:10.3389/fmicb.2022.1017773)
Supplement: Supplementary file 1 [file Data_Sheet_1.docx]

Supplementary Material

# Supplementary Figures and Tables

## Supplementary Figures


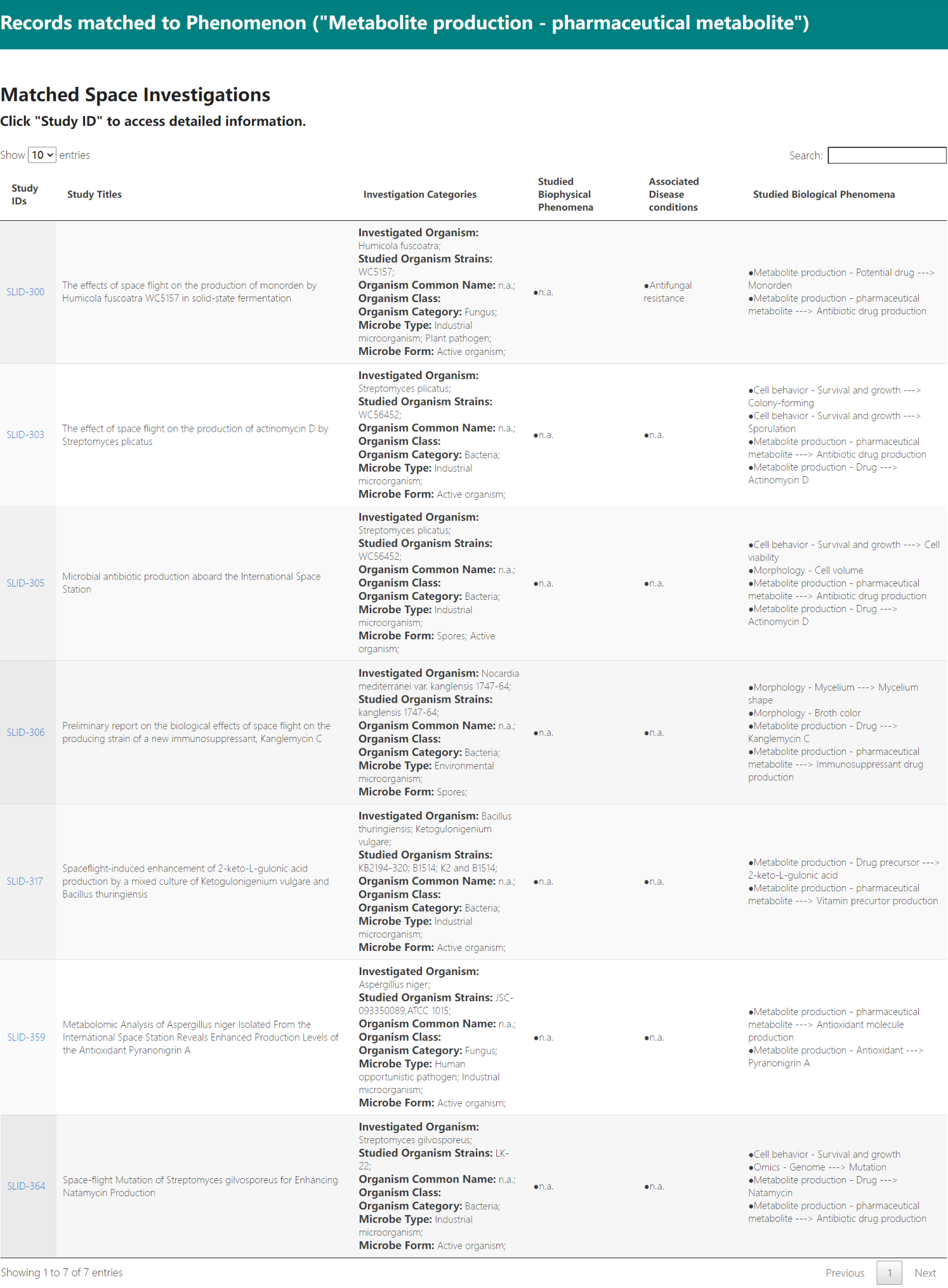


**Supplementary Figure 1.** The browse result of the tag “Metabolite production - pharmaceutical metabolite”.

## Supplementary Tables

**Supplementary Table 1.** Abbreviation list

| Abbreviation | Definition |
| --- | --- |
| AI | Artificial Intelligence |
| AMR | Antimicrobial Resistance |
| AU | Approximately Unbiased |
| CGBA | Commercial Generic Bioprocessing Apparatus |
| EEA | European Space Agency |
| ES | Enrichment Score |
| GSEA | Gene Set Enrichment Analysis |
| ISS | International Space Station |
| JAXA | Japan Aerospace Exploration Agency |
| LSDA | Life Science Data Archive |
| MOBIAS | Multiple Orbital Bioreactor with Instrumentation and Automated Sampling |
| NASA | National Aeronautics and Space Administration |
| RPM | Random Positioning Machine |
| RWV | Rotating Wall Vessel |
| SLIs | Spaceflight life investigations |
| SpaceLID | Space Life Investigation Database |

**Supplementary Table 2.** Data categorization of SpaceLID and established space life science databases

| Databases | LSDA | EEA | GeneLab | SpaceLID |
| --- | --- | --- | --- | --- |
| Data categorization^*^ | **1. Research:** research fields, 43 items, e.g., Behavior and performance.  **2. Principal Investigator:** 1328 items.  **3. Species:** Investigated organisms in common names, 105 items, e.g., Human.  **4. Keyword:** Keywords of the reports, 1752 items.  **5. Program:** Type of study program, 16 items, e.g., Apollo.  **6. Mission:** Name of spaceflight or ground simulation missions, 333. items, e.g., Apollo 10.  **7. Experiment Type:** Project of experiments, 40 items, e.g., Advanced Human Support Technologies (AHST).  **8. Payload:** Payload ID of the experimental equipment and materials, 534 items.  **9. Center:** The institution that performed the experiment, 10 items.  **10. Alias:** Other names or IDs of the experiments.  **11. Mission Launch Year:** 1958-2022 | **1. Platforms/Facilities:** Aircrafts or other vehicles used in the investigation, 6 items, e.g., Space Stations.  **2. Missions/Campaigns:** Spaceflight or simulation missions, 360 items, e.g., 1st ESA Parabolic Flight Campaign.  **3. Country:** Country participated in the experiments, 46 items  **4. Research Areas:** Including flowing two categories:  **4.1 Physical Sciences:** 67 items, e.g., Aggregation Phenomena.  **4.2 Life Sciences:** 25 items e.g., Animal Physiology.  **5. Period:** 1972-2023 | **1. Data Source:** Source of omics data, 4 items, e.g., NIH GEO.  **2. Project Type:** Ground, Spaceflight, Parabolic Flight Study.  **3. Assay Type:** Investigation methods, 16 items, e.g., Microarray.  **4. Factor:** Experiment of mission parameters, 30 items, e.g., Genotype.  **5. Tissue:** Investigated materials, 90 items, e.g., Spores.  **6. Organism:** Investigated organism or organism categories in common names, 14 items, e.g., Bacteria. | **1. Microbe:** The investigated microbes, provide the following categorization information:  **1.1 Microbe Class:** The scientific and common names (if available) of the organisms, classified by their relation to human, 14 classes, e.g., Human pathogen.  **1.2 Microbe Form:** Four kinds of microbe form, e.g., Active organism.  **1.3 Microbe Profile:** Investigated objectives, 36 items, e.g., Biofilm.  **1.4 Disease/Health Condition:** Associated diseases or conditions of the investigation, 5 items, e.g., Antibiotic resistance.  **2. Human:** Human investigations, provide the following categorization information:  **2.1 Human Part:** Biospecimen used in the investigation, 20 items, e.g., Blood.  **2.2 Human Profile:** Investigated objectives, 32 items, e.g., Blood components.  **2.3 Disease/Health Condition:** Associated diseases or conditions of the investigation, 32 items, e.g., Anemia.  **3. Animal:** Animal investigations, provide the following categorization information:  **3.1 Animal Class:** The scientific and common names of the organisms, classified by taxon, e.g., Amphibian.  **3.2 Animal Part:** Biospecimen used in the investigation, 54 items, e.g., Whole animal.  **3.3 Animal Profile:** Investigated objectives, 44 items, e.g., DNA damage.  **3.4 Disease/Health Condition:** Associated diseases or conditions of the investigation, 30 items, e.g., Behavioral abnormalities.  **4. Plant:** Plant investigations, provide the following categorization information:  **4.1 Plant Class:** The scientific and common names (if available) of the organisms, classified by their relation to human, 4 classes, e.g., Food.  **4.2 Plant Stage:** 7 items, e.g., Seed to seed.  **4.3 Plant Part:** Biospecimen used in the investigation, 21 items, e.g., Leaf.  **4.4 Plant Profile:** Investigated objectives, 39 items, e.g., Yield.  **5. Project Type:** Class of the aircrafts used in the investigation, 6 items, e.g., Space Station; or Ground simulation studies.  **6. Investigated Time:** 1973-2022. |
